# Food choice, activity level, and carbon footprint: exploring potential for sustainable food consumption practices in young adults

**DOI:** 10.3389/fnut.2024.1449054

**Published:** 2024-08-20

**Authors:** Mari Wollmar, Anna Post, Agneta Sjöberg

**Affiliations:** Department of Food and Nutrition and Sport Science, University of Gothenburg, Gothenburg, Sweden

**Keywords:** plant-based, protein intake, climate impact, sports nutrition, sustainability, sustainable diets

## Abstract

This study aims to explore climate consciousness in relation to food consumption in young adults, examining its relationship with physical activity level and gender. A mixed-method approach is utilized, integrating seven-day food records and semi-structured interviews, employing social practice theory in our analytical framework. Our cohort of 47 participants (25 women, 22 men) displays varied diets, from omnivores to vegans. Moderately-active women show the lowest carbon footprint, favoring climate-conscious choices related to lower energy needs and plant-based preferences. Highly-active individuals consume more energy, resulting in a higher carbon footprint. Gender differences are evident, women were inclined to climate-conscious food practices motivated by animal ethics and health concerns. Conversely, men demonstrated a tendency for meat consumption. Participants share an understanding of carbon footprint, reflecting a solid awareness of food-related climate impact but differ in priorities; performance for highly-active, and economy for moderately-active. This highlights a mix of commonalities and distinctions, informing flexible, sustainable food practices. Higher activity levels are linked to greater energy needs and a higher carbon footprint. Moderately-active women show the most climate-conscious food choices, leading to the lowest carbon footprint. Our findings indicate that highly-active individuals and men have significant potential to improve climate-adapted food consumption.

## 1 Introduction

According to the Intergovernmental Panel on Climate Change (IPCC), the food system is responsible for approximately 25–30% of global greenhouse gas emissions (carbon footprint) (1). The 2030 Agenda for Sustainable Development emphasizes sustainable practices and climate-resilient food systems to mitigate and adapt to climate change impacts (2). Further, the European Green Deal encourage the consumption of healthier and more sustainable food options, such as plant-based diets, to reduce the environmental footprint of food consumption (3). Therefore, to move forward, it is recommended to shift from animal protein sources, with a high amount of CO_2_e per kg such as beef and pork (4), to plant-based alternatives, such as increased intake of legumes and grains (5, 6). No single metric can capture all environmental effects of foods. For food and diet studies, carbon footprint is usually recommended, often presented as carbon dioxide equivalents (CO_2_e) (7). In Sweden, the average carbon footprint from food consumption was 1.45-ton CO_2_e per year in 2021 (8). This footprint has been steadily declining, reaching its lowest level in 2020 at 1.42-ton CO_2_e (8). Further, the embrace of plant-based diets is on the rise, showing a continuous increase inflexitarians for the sixth consecutive year (2015–2021) and a noteworthy change in societal acceptance of vegetarian food (9). Exploring connections between environmental factors and health considerations suggests that embracing these synergies could be a compelling driver for sustainable food consumption (10). Food consumption may also rely on preferences and cognitive strategies that help manage conflicting priorities. For example, health motivations may overlap with sustainable activities, and successful ecolabels often combine sustainability attributes with health benefits (11, 12). Moving toward sustainable food consumption requires understanding dietary habits and mundane routines. A study on reducing meat consumption found diverse motivations, from health to ethical concerns, with flexitarians opting for plant-based alternatives or smaller meat portions (13).

Factors contributing to sustainability include gender and education level (10). Age and gender play crucial roles in embracing plant-based protein sources, with young women showing the most enthusiasm (14–16). Conversely, research on male athletes suggests a shifting perception of diets away from traditional notions of meat and masculinity (17).

Dietary habits in physically in peoples with physical activity often contradict the suggestion that reducing energy intake and energy-dense foods are essential for achieving sustainable diets (18). Our previous study on recreational athletes suggests that their food practices prioritized performance and health over sustainability. In addition, the study revealed an awareness of food’s climate impact, where sustainability was important as long as it did not affect performance (19).

Social practice theories, exemplified by Marshall (20) and Spendrup and Hovmalm (16), offer a nuanced understanding of group dynamics, highlighting the adaptability of food consumption in supporting sustainable transformations and revealing gender differences in plant-based food consumption. This perspective serves as a comprehensive framework for evaluating the incorporation of sustainability into dietary practices (21).

Social practice theories feature the importance of considering the cultural and historical context of our actions and experiences. Further, social practice theories are not fixed but are subject to change and transformation (22). According to Shove et al. (23), a person’s ability to participate in a specific practice is referred to as competence. A practice includes cognitive skills and using physical material elements such as tools, infrastructure, and even the body. These elements must have social and symbolic significance to form a practice encompassing emotions, thoughts, and motivations and provide meaning.

In this study, food consumption is understood as a dynamic and ongoing process shared by people with similar circumstances and common interests. Social practice theories propose that human actions are rooted in norms rather than pure rationality, allowing flexibility in social and cultural expressions (20, 24).

This study aims to explore climate consciousness in relation to food consumption in a sample of young Swedish adults, examining its relationship with physical activity level and gender. The following research questions were asked: How can the relationship between food consumption, activity levels, and gender be understood in terms of energy intake, carbon footprint, and protein sources? How do elements of food consumption influence the carbon footprint? How is sustainability considered within the context of food consumption?

## 2 Materials and methods

This study uses a mixed-method approach based on interviews and data collected from the methodological project Measuring Energy Expenditure and Dietary Intake at Different Activity Levels (MEDAL). A seven-day food record was performed to provide a detailed picture of the participants’ food intake. The quantitative data of food records add information about protein sources, food intake, and carbon footprint expressed as CO_2_e (25).

Participants were recruited through posters on university bulletin boards, emails to students at the Faculty of Education at Gothenburg University, social media, and personal contacts with running and soccer clubs between October 2020 and April 2021. The inclusion criteria were women and men aged 18–40 with different activity levels, complete inclusion and exclusion criteria for the MEDAL study can be found in Supplementary Table 1. All different types of dietary preferences were welcome to participate. The categorization for Moderate-to-vigorous physical activity (MVPA) used the World Health Organization 2020 guidelines on physical activity and sedentary behavior (26) to identify different activity levels. The study sought 20 participants in two groups: one with up to 150 min of MVPA per week and another with at least 300 min of MVPA per week, evenly split between men and women. Resting energy expenditure (RMR) was measured using indirect calorimetry (27), described in detail elsewhere (28).

This study was approved by the Gothenburg Regional Ethics Committee #2019-05316. Before signing the consent form, participants were provided oral and written information. Participants were free to drop out of the study without further explanation. The study used identification numbers to protect the participants’ identities.

### 2.1 Data collection

Data were collected through semi-structured interviews and a seven-day food record. All participants were interviewed in connection with completing the food record. The interview guide comprised 12 open-ended, non-leading questions that explored several key areas: food shopping habits, dietary preferences, dietary changes over time, the relationship between diet and health, and considerations regarding the climate impact of food choices. The interviews were held at the Center for Health and Performance at University of Gothenburg and lasted 20–60 min. Compliant with the aim, the interviews focused on elements related to the carbon footprint of food. However, the participants were informed that the interview would address their food consumption but not that climate consideration was an area of interest to avoid influencing participants’ responses and social-desirability bias. An interview guide provided structure, but the questions were open-ended, offering flexibility and undirected answers. The interviews were transcribed in verbatim.

The interview protocol was designed to explore participants’ food choices, focusing on the coordination of activities, the stability and repetition of behaviors, the influence of contextual factors, and the dynamic nature of their practices. According to Shove et al. (23), a person’s ability to participate in a specific practice is referred to as competence. A practice includes cognitive skills and using physical material elements such as tools, infrastructure, and even the body. These elements must have social and symbolic significance to form a practice encompassing emotions, thoughts, and motivations and provide meaning.

Each participant performed a seven-day weighed food record using the online tool Nutrition Data (Nutrition Data Sweden AB, Norsjö., Sweden) version 20.11.11. Each participant was offered a portable scale for weighing food. Participants were encouraged to upload pictures in the Nutrition Data app to clarify their intake. The messaging function was used to send standardized encouraging messages on days three and six to increase compliance. To assess the accuracy of the reported energy intake, it was divided by RMR, and values below 1.2 are considered low energy intake (29).

Further, the RISE climate database (30) in DietistNet Kost och Näringsdata, 2020 version 1.6 was used to assess CO_2_e. Finally, protein sources were divided into animal- or plant-based by manually evaluating the food records.

### 2.2 Data analysis

The results include descriptive statistics as a complement to the interview findings. The normality of all variables was evaluated using the Shapiro-Wilks test. The correlation was tested using Pearson’s correlation. A student’s t-test and analysis of variance (ANOVA) were used to compare activity levels and gender. In cases where significant results were obtained in the ANOVA, the Bonferroni post-hoc test was conducted to correct for multiple tests. *p*-values of 0.05 or lower were considered significant. SPSS Statistics: Version 29.0.0.0 (241) (31) was used for all statistical analyses.

**Figure 1:**
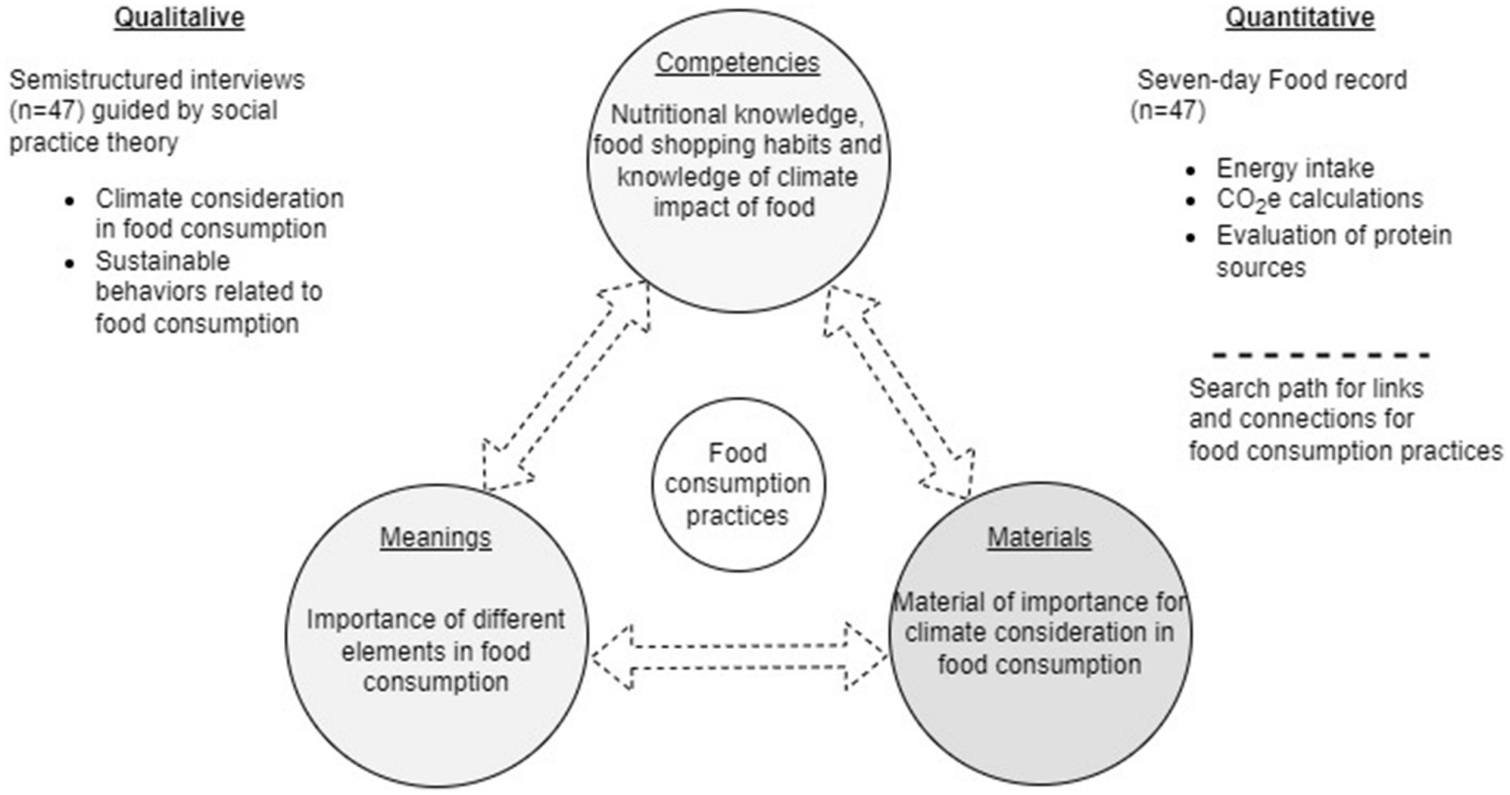
A methodological framework for combining quantitative findings from the food records and the elements; competence, material, and meaning.

Qualitative understandings are triangulated with quantitative data from food records to provide a comprehensive and nuanced analysis. Inspired by social practice theory, the analysis primarily centered on ‘sayings’ in the interviews related to climate impact and sustainability in the context of food consumption and ‘doings’ corroborated by the food records (Figure 1). The term “climate-conscious” will refer to considering carbon footprint within sustainability, while “sustainable” will encompass a broader perspective such as health, economy, and animal welfare. The perspective described by Shove et al. (23) was employed throughout the analysis to interpret and contextualize findings.

The analysis of the interviews was conducted separately by the first and second authors and was subsequently discussed to establish consensus. The authors’ curiosity about attitudes toward climate considerations within food consumption motivated the study.

## 3 Findings

### 3.1 Participant profiles and dietary dynamics

A total of 47 participants, 25 women and 22 men completed the study protocol. Among the participants, the moderately-active group comprised 14 women and eight men, whereas the highly-active group comprised 11 women and 14 men. Detailed information about the subject characteristics and socio-demographics can be found in Table 1 and Supplementary Table 2. The average age of the participants was 28.6 years. Among the participants, 51% identified as omnivores, while 49% identified as flexitarian, pescetarian, vegetarian, or vegan. Among the omnivores, the majority (71%) were found in the highly-active group. Conversely, in the moderately-active group, 65% followed diets other than omnivore.

**Table 1:** Subject characteristics.

	All (*n* = 47)	Moderately-active (*n* = 22)	Highly-active (*n* = 25)		Women (*n* = 25)	Men (*n* = 22)	
	Mean (SD)	Mean (SD)	Mean (SD)	*p*-value*	Mean (SD)	Mean (SD)	*p*-value*
Age (years)	28.62 (5.41)	27.55 (5.38)	29.56 (5.36)	0.206	27.88 (5.27)	29.45 (5.55)	0.325
Weight (kg)	72.69 (15.56)	76.03 (20.86)	69.76 (8.02)	0.170	66.65 (10.76)	79.56 (17.47)	0.003*
Height (cm)	175.50 (0.10)	172.64 (0.11)	178.02 (0.09)	0.066	168.26 (7.22)	183.78 (5.30)	<0.001*
BMI (kg/m^2^)	23.56 (4.23)	25.32 (5.36)	22.01 (1.95)	0.011*	23.54 (3.59)	23.57 (4.94)	0.982
RMR (kcal/d)	1,503 (269)	1,497 (348)	1,507.08 (254.74)	0.911	1,354 (210)	1,671 (299)	<0.001*
EI/RMR	1.78 (0.42)	1.56 (0.28)	1.97 (0.44)	<0.001*	1.66 (0.41)	1.91 (0.40)	0.042*

BMI, Body Mass Index; RMR, Resting Metabolic Rate; kcal, kilo calories; AEE, Activity Energy Expenditure; EI, Energy Intake. Data are presented as mean (standard deviation). *Comparisons between groups tested with student’s t-test, *p*-values < 0.05 was considered significant.

A significant correlation was observed between energy intake and kg CO_2_e per day, with a Pearson coefficient of 0.4. The mean protein intake was 99.9 g/d; animal-based proteins comprised 56% of the total protein intake. The protein intake (g/d) and kg CO_2_e/d exhibited a strong and significant correlation, with a Pearson coefficient of 0.5. Grains and dairy were the two food groups that contributed the most to protein intake, with 25 and 22%, respectively.

### 3.2 Decoding gender dynamics in food practices

The carbon footprint differences between women and men highlight food choices as a material aspect of food consumption practices. By calculating carbon footprints from dietary records, we can quantifiably measure the climate impact, with variations attributed to both food types and quantities consumed. Women’s mean energy intake was notably lower than men’s, at 9.2 MJ/d versus 13.1 MJ/d, respectively, correlating with a lower carbon footprint. Moderately-active women exhibited the smallest footprint at 15.4 kg CO_2_e weekly, contrasting with moderately-active men at 36.4 kg CO_2_e, highly-active men at 30.8 kg CO_2_e, and highly-active women at 27.3 kg CO_2_e daily.

Interviews revealed similar understandings and considerations among women and men regarding various factors impacting carbon footprints, including reduced consumption of red meat and adoption of vegetarian alternatives. This was evident in their statements and actions, as reflected in their seven-day food records, where nearly all participants reported consuming at least one vegetarian meal. Women showed a stronger inclination toward considering animal ethics in meat consumption decisions, leading to significantly lower red meat intake compared to men. Both climate awareness and animal ethics influenced meat consumption choices, with only 40% of women following an omnivore diet. At the same time, the majority prioritized climate-conscious options like flexitarianism, pescetarianism, vegetarianism, or veganism. On the other hand, the lower intake of grains among female participants is not related to climate considerations but rather emphasis on weight management. Meat and their vegetable counterparts (for example, meat analogs) represent material elements of the food practice.

In contrast, among men, the majority (64%) were omnivores, displaying a different dietary pattern than women. The dietary differences between men and women highlight food items as elements of their food practice. Men’s experiences of reducing meat consumption at home revealed situational influences on dietary choices within the household context, where cooking to accommodate a vegetarian partner for the sake of peace provided meaning. Men living with a vegetarian female partner indicated that reducing meat consumption at home became more accessible since their partner abstained from meat. This finding suggests that women in the household often initiated a decrease in meat consumption, even among men. More detailed information on energy intake, macronutrients, carbon footprint and preferred diets can be found in Table 2 and Supplementary Table 1.

**Table 2:** Total energy intake, macronutrient distribution, and carbon dioxide equivalents.

	All (*n* = 47)	Moderately-active (*n* = 22)	Highly-active (*n* = 25)		Women (*n* = 25)	Men (*n* = 22)	
	Mean (SD)	Mean (SD)	Mean (SD)	*p*-value	Mean (SD)	Mean (SD)	*p*-value
Total energy intake (kcal)	2,635 (659)	2,303 (115)	2,928 (125)	<0.001*	2,204 (389)	3,125 (554)	<0.001*
Total energy intake (MJ)	11.0 (2.8)	9.6 (0.5)	12.3 (0.5)	<0.001*	9.2 (1.6)	13.1 (2.3)	<0.001*
Protein intake (g/d)	99.9 (32.2)	76.9 (18.3)	120.2 (29.2)	<0.001*	90.4 (34.1)	110.7 (28.1)	0.032*
Protein g/kg body weight	1.4 (0.5)	1.1 (0.5)	1.8 (0.1)	<0.001*	1.4 (0.6)	1.5 (0.4)	0.765
Protein E%	15.6 (4.9)	13.6 (2.4)	17.4 (5.8)	0.006*	14.5 (2.6)	16.9 (6.4)	0.118
Carbohydrate intake (g/d)	286.6 (76.4)	243.6 (12.7)	324.4 (14.0)	<0.001*	244.1 (51.1)	335.0 (72.0)	<0.001*
Carbohydrate E%	46.0 (4.5)	45.0 (5.6)	46.9 (3.9)	0.165	46.5 (4.5)	45.4 (5.2)	0.436
Fat intake (g/d)	108.0 (36.9)	100.2 (7.0)	114.8 (7.9)	0.178	85.9 (24.8)	133.1 (32.1)	<0.001*
Fat E%	36.7 (6.2)	39.2 (4.8)	34.5 (6.6)	0.008*	37.6 (5.0)	35.8 (7.3)	0.326
Fiber intake (g/d)	31.4 (10.6)	27.1 (2.5)	35.2 (1.6)	0.008*	29.8 (10.3)	33.2 (11.0)	0.287
Fiber (g/MJ)	2.9 (0.8)	2.8 (1.0)	2.9 (0.7)	0.528	3.2 (0.9)	2.5 (0.5)	0.002*
Co_2_e/day (kg)	3.8 (1.8)	3.3 (0.5)	4.2 (0.2)	0.114	3.0 (1.2)	4.7 (2.0)	<0.001*
Co_2_e/MJ (kg)	0.35 (0.15)	0.34 (0.04)	0.35 (0.02)	0.868	0.33 (0.12)	0.37 (0.18)	0.300

MJ, Mega Joule; kcal, kilo calories; Co_2_e, carbon dioxide equivalent; E%, energy percentage of total intake, 1.8E% from alcohol. *Comparisons between groups tested with student’s t-test, p-values < 0.05 was considered significant.

Awareness of the carbon footprint of various foods, particularly red meat, was evident, with respondents noting increased consumption of vegetarian meals and plant-based protein alternatives over the past decade. Actions aligned with statements reflect a societal shift toward accepting vegetarianism. This transformation carries implications for promoting climate-conscious and sustainable dietary practices.

### 3.3 Energy intake and carbon footprint in relation to activity level

The highly-active participants, with a mean energy intake of 12.3 MJ, prioritized maintaining energy levels for extensive training sessions, significantly higher than the 9.6 MJ intake of the moderately-active group. This difference was notable, particularly between moderately-active women and both moderately-active and highly-active men. Correspondingly, the carbon footprint was higher in the highly-active group (4.2 kg CO_2_e) compared to the moderately-active group (3.3 kg CO_2_e).

Moreover, the highly-active group showed significantly elevated intakes of carbohydrates, protein, and fiber compared to the moderately-active group, indicating a focus on sustaining energy levels for prolonged training sessions, a priority less emphasized by the moderately-active group. On an annual scale, the highly-active group’s carbon footprint was estimated at 1.53 tons of CO_2_e (daily CO_2_e multiplied by 365 days), while the moderately-active group had a footprint of 1.20 tons of CO_2_e.

Despite being recreational athletes, highly-active participants in the study expressed a strong desire to mirror the dietary practices of professional athletes, drawing inspiration from their meticulous planning and specific food choices. The emphasis on acquiring sufficient energy for prolonged training sessions reflects a distinct competence in optimizing performance through dietary practices. This desire to emulate professional athletes suggests a competence influenced by external factors and aspirational goals. The focus on performance was further evident in the significantly higher protein intake, with the highly-active group consuming 1.8 g per kilogram of body weight per day compared to 1.1 g in the moderately-active group. Highly-active females had the highest intake at 1.9 g/kg body weight per day. Additionally, the highly-active group showed significantly elevated consumption of dairy and whey protein sources, highlighting their preference for protein-rich foods compared to the moderately-active group. Comparisons between group and gender can be found in Supplementary Table 3.

The focus on performance in the highly-active group increased their carbon footprint mainly through a high caloric intake and animal-based protein sources such as dairy and whey protein products. The highly-active group with 68% of participants identified as omnivores contrast with the moderately-active group, where only 32% follow an omnivorous diet. Detailed protein source information can be found in Table 3, with preferred diets outlined in Supplementary Table 2. The higher intakes of carbohydrates, protein, and fiber in the highly-active group signify a material aspect of their food practices. The emphasis on protein-rich foods, particularly dairy and whey, aligns with the focus on sources supporting athletic performance. The significant differences in protein intake and sources highlight the tangible aspects of their dietary choices, influenced by their activity level and performance-oriented goals. The meaning associated with food practices in the highly-active group is multifaceted and conflicting. The carbon footprint is, in this context, negatively influenced by pursuing specific meanings, such as achieving optimal athletic performance. The participants’ statements and choices illustrate how meanings associated with food extend beyond nutrition to include performance goals and mimicking dietary choices made by professional athletes.

**Table 3:** Protein intake and protein sources (g/day).

	All (*n* = 47)		Moderately-active (*n* = 22)	Highly-active (*n* = 25)	*p*-value	Women (*n* = 25)	Men (*n* = 22)	*p*-value
	Mean (SD)	%	Mean (SD)	Mean (SD)		Mean (SD)	Mean (SD)	
Protein sources								
Plant-based	43.7 (15.6)	44	35.8 (14.7)	50.7 (13.1)	<0.001*	38.9 (13.2)	49.2 (16.7)	0.023*
Animal-based	55.5 (28.6)	56	41.1 (16.8)	68.2 (31.0)	<0.001*	50.5 (32.0)	61.2 (23.5)	0.206
Red meat	10.8 (12.1)	10.8	9.8 (14.6)	11.7 (9.7)	0.591	6.1 (8.0)	16.1 (13.9)	0.006*
Poultry	7.8 (11.32)	7.8	4.6 (6.3)	10.6 (13.9)	0.061	8.8 (14.3)	6.6 (6.8)	0.505
Fish and seafood	6.3 (7.0)	6.3	4.8 (6.3)	7.6 (7.4)	0.170	5.6 (7.5)	7.0 (6.4)	0.476
Dairy	21.9 (12.2)	22.0	16.9 (7.9)	26.5 (13.7)	0.005*	20.6 (10.7)	23.6 (13.8)	0.406
Egg	2.3 (2.7)	2.3	2.8 (3.1)	1.9 (2.2)	0.270	2.2 (2.7)	2.4 (2.7)	0.841
Whey products	5.7 (10.6)	5.7	0.8 (1.9)	10.0 (13.1)	0.002*	6.6 (11.5)	4.5 (9.6)	0.500
Grains	24.6 (8.6)	24.6	20.1 (7.2)	28.7 (7.8)	<0.001*	20.0 (6.2)	29.8 (8.1)	<0.001*
Legumes	2.6 (3.3)	2.6	2.6 (3.5)	2.5 (3.2)	0.966	3.0 (3.5)	2.0 (3.1)	0.285
PBMAs	5.7 (7.6)	5.7	3.6 (4.8)	7.5 (9.1)	0.073	6.0 (8.1)	5.4 (7.2)	0.799
Vegetables	2.9 (1.5)	2.9	2.6 (1.3)	3.1 (1.6)	0.239	2.8 (1.4)	3.0 (1.5)	0.706
Root vegetables	2.1 (1.5)	2.1	2.3 (1.8)	1.9 (1.3)	0.307	1.8 (1.7)	2.3 (1.3)	0.270
Fruits and berries	1.5 (1.0)	1.5	1.2 (0.8)	1.8 (1.2)	0.045*	1.6 (0.8)	1.5 (1.3)	0.898
Nuts and seeds	4.4 (4.6)	4.4	3.4 (3.8)	5.2 (5.2)	0.185	3.7 (4.0)	5.2 (5.2)	0.277
Other	0.6 (0.7)	1.0	0.5 (0.5)	0.8 (0.7)	0.079	0.5 (0.6)	0.7 (0.8)	0.115

Data are presented as mean (standard deviation) Daily intake in %. The protein source “other” is from uncategorised candy and drinks with branched chained amino acids (BCAA). *Comparisons between groups tested with student’s t-test, *p*-values < 0.05 was considered significant.

### 3.4 Woven perspectives: merging interviews and food records for a comprehensive insight into food practices

The interviews were analyzed for elements related to competence, material, and meaning. All participants demonstrated climate awareness in their food consumption practices, revealing a nuanced understanding of the environmental impact of different food choices. Their knowledge of which foods contribute to high or low carbon footprints underscores their awareness of the environmental consequences of their dietary decisions. This awareness was evident in their sayings, where they commonly acknowledged that meat products have a high carbon footprint, while vegetables, fish, poultry, and dairy products have a lower impact.

Participants also exhibited competence in considering vegetarian alternatives to animal protein sources in their meals. However, while many expressed interests in eco-labels and locally produced goods, their actions were often hindered by perceived high costs. Competence was evident in recognizing the environmental significance of choosing vegetarian protein sources over animal-based alternatives, particularly meat products. However, this competence was not consistently applied when replacing meat with dairy products, such as cheese and whey, revealing a gap in understanding.

Organizing and structuring food purchases and cooking are key components of food practices, and differences are observed between activity groups. Material elements, such as lunch boxes, play a crucial role in how participants organize their food practices. In the highly-active group, there is a focus on energy for training, which is reflected in the tangible materiality of lunch boxes and dietary choices. Conversely, economic factors and shared living arrangements influence the organizational aspects of the moderately-active group. Household dynamics, including the presence of other members, impact routines related to meal planning, cooking, and consumption habits. Additionally, economic constraints, such as higher prices for meat and fish combined with low income, as well as considerations of frozen versus fresh goods, influence food practices.

Climate awareness has materialized in homemade lunch boxes, which, combined with proper competence foster a climate-conscious food practice. Cooking in advance also leads to rational food purchases and less food waste. The foods are essential materials; choosing foods with low carbon footprint requires competence and could provide meaning.

Furthermore, economy appear as highly influential among low-income students. Budget constraints significantly influence food consumption, underscoring the impact of financial factors on sustainable food practices. The moderately-active group, containing a larger proportion of university students compared to the highly-active group, appears particularly influenced by economic concerns. However, also in the highly-active group, students show similar economic considerations.

Ethical concerns, particularly regarding animal welfare, serve as a driving force behind the reduction in meat consumption, especially among moderately-active women. This highlights that the meaning attached to food choices extends beyond environmental considerations to encompass ethical perspectives. Participants viewed vegetarian foods as environmentally friendly, healthy, and sustainable, aligning with their goals for personal well-being and longevity. Preference was given to Fairtrade-labeled products whenever available and affordable.

While Swedish meat was valued for its perceived safety, low antibiotic usage, and preference over imported meats, it was not purchase frequently. Instead, consumers often opted for poultry or vegetarian alternatives. These sentiments were echoed in the food records, underscoring the diverse considerations and perspectives that contribute to sustainable food practices.

**Figure 2:**
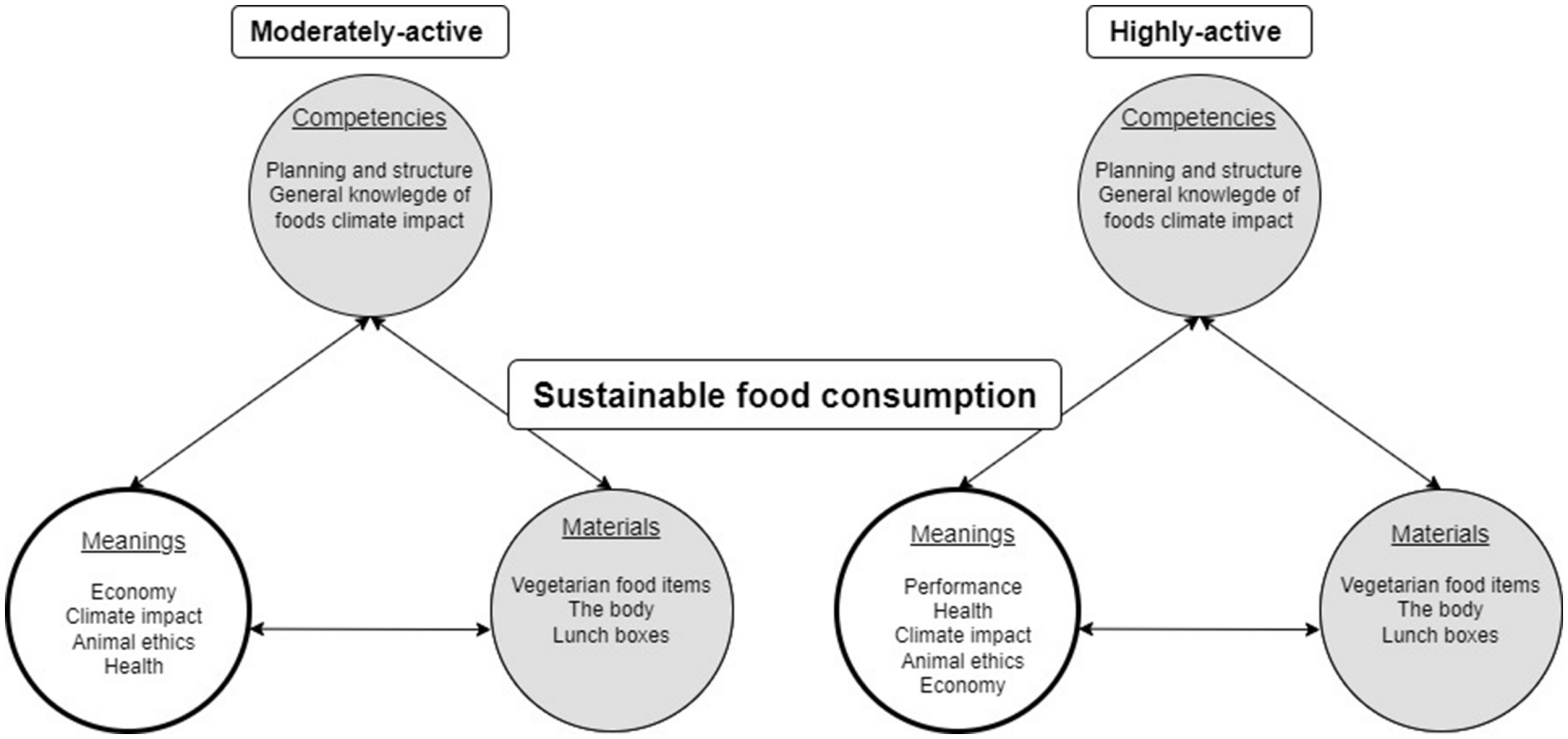
Visualizing the shared understanding of elements of competence and material of importance for food-related carbon footprint. Both groups share these fundamentals but differ in meaning-bearing elements.

The primary difference between the groups lies in meaning-bearing elements, where both groups find meaning in being climate-conscious, albeit with variations. The highly-active group values performance, while the moderately-active group emphasizes economic considerations. The resulting sustainable food practices, depicted in Figure 2, illustrate a blend of shared intentions and distinctions.

## 4 Discussion

Reflecting the principles of social practice theory, this section explores the connections between gender, activity level, and the adoption of sustainable food practices. In Figure 2, we display the food practices found by triangulating data from the seven-day food records and the social practice theory-inspired analysis. We start by discussing the meaning-bearing elements in which the most significant discrepancies were found.

### 4.1 Elements in a sustainable food practice

The two activity groups exhibit distinct meanings attached to their food choices. In the moderately-active group, priorities revolve around animal ethics and economic considerations, while the highly-active group places greater emphasis on performance and health. These differing value systems emphasize the importance of products that address both animal welfare and health concerns. It is not uncommon for products to combine various value statements, as noted by Waldman et al. (11), who describe eco-labels that promote health benefits as part of their marketing strategy.

Although the consideration of the carbon footprint may not be overtly emphasized, its influence subtly emerges, especially in discussions comparing meat and vegetable protein sources. To gain a comprehensive understanding of food consumption practices, it is crucial to acknowledge and recognize all elements that contribute to meaning-making, including health, performance, ethics, and economics (23). The creation of meaning in sustainable food consumption practices often involves negotiation among various viewpoints, including considerations of performance and economy, even when the overarching goal is to eat sustainably. Allowing different elements to take precedence at different times may be necessary to sustain the practice. Marshall (20) describes this flexibility as essential for maintaining sustainable food consumption practices.

The level of knowledge regarding the climate impact of food in our study reflects what can be understood as ‘common knowledge’ shaped by media and social media influences. The idea that social media significantly contributes to enhancing consumers’ intentions to consume products with lower carbon footprint is supported by the findings of Rini et al. (32).

### 4.2 Energy intake, body dimensions, and carbon footprint

Engaging in structured and well-planned grocery shopping and organizing menus for weekdays and lunch boxes minimizes food waste and aligns with climate-conscious food consumption practices (19, 20, 33). These practices involve thoughtful and intentional shopping and meal preparation approaches, which can contribute to reducing domestic food waste (33).

Further, vegetarians and flexitarians in both activity groups replaced meat with cheese as a protein source. However, cheese accounts for a significant carbon footprint, comparable to chicken, pork, fish and egg (34). The unreflective way to use cheese displays that knowledge concerning the carbon footprint of different food groups is varied and often divided into beef versus vegetables. Interestingly, when discussing dairy products, the participants did not mention carbon footprint or animal welfare in the same way as meat. This selectivity highlights that practices are not fixed but shaped by contextual factors (21, 23), and certain elements may be more salient or visible in the participants’ collective understanding, influenced by media narratives (32) and societal discussions.

The findings suggest that both activity level and gender play significant roles in influencing carbon footprint. Individuals with shorter stature or lower weight and muscle mass naturally tend to have a lower carbon footprint compared to taller or heavier individuals when their food intake aligns with their energy requirements. Given that women typically have smaller body dimensions and lower muscle mass, their energy needs are generally lower, facilitating their ability to achieve a lower carbon footprint than men. However, as gender and height are inherent factors, men must make even more selective food choices to keep their carbon footprint low. The combination of a lower energy intake and the choice of plant-based foods results in a successful combination regarding carbon footprint in moderately-active women compared to other groups. These findings are consistently observed in multiple studies, indicating that women tend to have a lower carbon footprint than men (10, 35, 36). Concurrently, our study shows a markedly lower carbon footprint among the female participants. However, this difference was diminished when adjusting for energy intake, affirming that inherent factors play a significant role in food intake.

### 4.3 Vegetarianism, domestic trends, and sustainable eating

There is often an association between skepticism about adopting vegetarian lifestyles and traditional notions of masculinity, particularly in the context of meat consumption (15). However, societal attitudes are shifting, particularly among young, educated men in urban areas. This population is increasingly receptive to integrating vegetarian elements into their food consumption, displaying a favorable outlook on dietary changes (18). Male participants in our study embrace vegetarian alternatives and express readiness to further reduce meat consumption if palatable alternatives are available. Food practices change continuously (24), and society’s widespread tolerance of vegetarianism affects eating practices, especially among young consumers (35).

Our study participants are part of a social context, young educated and living in a larger city, where vegetarian meals and the concern for climate change is present. Male participants are here rarely questioned about their choice of vegetarian options, a challenge previously faced by male consumers (18).

### 4.4 The influences of the higher education context on sustainable food practices

Another notable aspect of our study is the prevalence of university students, 53% of the participants. This demographic highlights the distinctive influence of the student lifestyle and financial considerations on food choices. Examining significant life moments reveals the transformative potential of major transitions (21). In this context, the transition to higher education emerges as a critical juncture, offering a unique opportunity to re-evaluate and reshape practices (37).

In Sweden, sustainability and climate action goals are incorporated into the higher education curriculum (38), adding another layer of complexity to our participants’ awareness. Keller et al. (21) argue that sustainable practices cannot be effectively implemented, adapted, or negotiated without considering the social context. They acknowledge that sustainable practices rely on social interaction and communication (21). The junction of our participant’s past exposure to climate-adapted school meals in primary education and the current integration of sustainability into higher education curricula might be a perfect opportunity to consolidate sustainable food practices.

### 4.5 Adopting a professional-like diet

The interviews reveal that the highly-active group uniquely values performance, a priority not shared by the moderately-active group. Female soccer players in the highly-active group focus on increasing muscle mass and staying lean, mirroring findings from a previous study of CrossFit athletes (19). These athletes emphasize protein intake, including whey protein and dairy products. In endurance sports like running, there is a common emphasis on maintaining a low body weight to enhance performance (39, 40). However, inadequate energy intake is prevalent among female athletes (41), posing a challenge in balancing a climate-conscious diet with health needs. Despite the higher protein intake requirements, it is possible to meet athletes’ nutritional needs while reducing climate impact (19, 42).

Professional athletes often serve as role models in various aspects, including food choices (43). This influence was evident in our study, where many highly-active participants drew inspiration from professional athletes’ diets. Despite not competing at a professional level, they believed that adopting such diets could enhance their performance. This focus on food as fuel can lead to increased energy intake and a higher carbon footprint, posing a challenge to reducing our overall carbon footprint from food. Therefore, it is crucial for athletes to meet their protein needs without exceeding them, utilizing flexitarian and plant-based strategies (42).

Promoting plant-based protein sources among professional athletes can influence recreational athletes and the broader population to adopt climate-conscious food practices. Studies show that sports celebrities, due to their visibility, status, and positive public image, effectively drive behavior change (44). Professional athletes, as influential role models, can significantly impact sustainable eating habits. Additionally, the athletic community requires evidence-based guidance that balances nutritional needs with climate goals.

### 4.6 Concerns and future research

Climate-conscious food consumption, as recommended by the EAT-Lancet, is linked to improved health (45). In our study, the primary motivation for moderately-active women to increase plant-based food intake was concern for animal welfare and personal health, resulting in the lowest carbon footprint. However, ensuring nutritional adequacy can be challenging with plant-based diets, especially for essential vitamins and minerals like B12, iron, calcium, and zinc (46). Women of fertile age have high iron requirements necessitating sufficient iron intake and bioavailability (47). Despite being more proactive in adapting their diets to climate concerns (14, 15), young women risk compromising their health if their diet is not well-balanced.

Our study raises several important questions: Should we establish a maximum daily carbon footprint for food consumption? Should it be a fixed value per individual, as proposed by initiatives like One Planet Plate (48) or should it be flexible based on energy requirements? Each approach has merits and drawbacks. A fixed recommendation, like those in One Planet Plate and EAT-Lancet, may be difficult to reach for groups with high energy needs. The EAT-Lancet diet is based on an average energy intake of 2,500 kcal per day and does not address varying energy requirements (6). This standard would be challenging for highly-active men with an average intake of 3,400 kcal per day. Should the carbon footprint be linked to an energy-adjusted system, which might be more attainable, or should it remain a fixed limit? Despite these considerations, the urgency to reduce the carbon footprint and combat climate change is unwavering.

## 5 Strengths and limitations

Combining interviews, food records, and carbon footprint calculations within the framework of social practice theory provides a robust and multi-dimensional approach. This synergy allows for a comprehensive examination of dietary practices. Integrating qualitative and quantitative data enhances the study’s validity and reliability. Qualitative insights offer depth and context, explaining the reasons behind dietary choices, while quantitative data provide measurable parameters like energy intake and carbon footprints to corroborate or challenge the interview findings.

A limitation is that over 50% of participants were university students, which is higher than the one-fourth of each age cohort attending higher education in Sweden. However, this might indicate future trends since higher education often drives societal development.

We used CO_2_e to evaluate the carbon footprint for better comparability across studies, although this metric does not cover all aspects of sustainable food consumption, such as biodiversity, land use, or organic food systems. Another strength was that the energy intake relative to resting metabolic rate (EI/RMR) aligned with appropriate levels for the participants’ activity levels, indicating that the online food record provided adequate food intake reporting at a group level.

## 6 Conclusion

In conclusion, our study explores climate consciousness among young adults, highlighting elements such as vegetarianism, lunch box usage, and weekly shopping habits. While all groups exhibit foundational sustainability principles, key distinctions emerge based on physical activity levels. Higher activity levels correlate with greater energy requirements and a higher carbon footprint. The highly-active group prioritizes performance in their food choices, while the moderately-active group focuses on economic considerations. Moderately-active women exhibit the most climate-conscious food choices, resulting in the lowest carbon footprint. Our findings suggest that groups with high physical activity levels and men have significant potential to contribute more to climate-adapted food consumption.

## Data availability statement

The datasets presented in this study can be found in online repositories. The names of the repository/repositories and accession number(s) can be found below on the Swedish National Data Service, https://snd.se/sv/catalogue/dataset/2024-413.

## Ethics statement

The studies involving humans were approved by the Gothenburg Regional Ethics Committee. The studies were conducted in accordance with the local legislation and institutional requirements. The participants provided their written informed consent to participate in this study.

## Author contributions

MW: Writing – review & editing, Writing – original draft, Visualization, Project administration, Methodology, Investigation, Formal analysis, Data curation, Conceptualization. AP: Supervision, Writing – review & editing, Writing – original draft, Visualization, Methodology, Formal analysis, Conceptualization. AS: Formal analysis, Writing – review & editing, Writing – original draft, Visualization, Supervision, Methodology, Conceptualization.
