# Supplementary material for: Food choice, activity level, and carbon footprint: exploring potential for sustainable food consumption practices in young adults
Source: Front Nutr. 2024 Aug 20;11:1449054. doi: 10.3389/fnut.2024.1449054 (PMC11370661; doi:10.3389/fnut.2024.1449054)
Supplement: Supplementary file 1 [file Table_1.DOCX]

| **Supplemental Table 1.** Inclusion and exclusion criteria | |
| --- | --- |
| **Inclusion criteria** | **Exclusion criteria** |
| - Women (50%) and men (50%)  18 – 40 year of age - The participant has ability to be compliant to the study protocol with regards to general health. - High active group at least 300 min of VPA - Low to medium active group no more than 150 min of VPA - Signed written informed consent. | - Female patients who are pregnant, lactating or have the desire to become pregnant during the study - Conditions that might affect BMR such as untreated or poorly controlled hypo- or hyperthyroidism - Other conditions that are hard to control for like poorly controlled diabetes, unstable heart disease, heart failure, recent stroke/TIA or other similar complications.      - Recent Covid-19 infection or suffering from post-covid symptoms - Artificial joints or metal elements in their body or wearing a pacemaker. - Not considered suitable if during assessment judged unable to comply to the study protocol. |

| **Supplemental Table 2.** Socio-demographics and Individual participant characteristics ranked by estimated yearly carbon footprint in tons of CO_2_e | | | | | | | | | | | |
| --- | --- | --- | --- | --- | --- | --- | --- | --- | --- | --- | --- |
|  |  |  |  |  |  |  |  |  |  |  |  |
| **Group/gender** (activity) | Age in years | Height in centimetres | Weight in kilograms | BMI | EI/RMR^1^ | Reported energy intake  in MJ/day | Estimated weekly carbon footprint in kg CO_2_e | Preferred diet | Main means of transportation when shopping for food | Occupation | Persons sharing household |
| **MA Female 1** | 32 | 167 | 51.0 | 18.3 | 1.57 | 8.1 | 9,5 | Vegetarian | Walk | Student | 2 |
| **MA Female 2** | 25 | 182 | 80.1 | 24.3 | 1.62 | 9.8 | 10,9 | Flexitarian | Walk | Student | 1 |
| **HA Female 1** (runner) | 30 | 166 | 62.0 | 22.6 | 1.46 | 8.6 | 11,6 | Vegan | Walk | Employed | 3 |
| **MA Female 3** | 29 | 169 | 58.1 | 20.5 | 2.29 | 8.9 | 12,4 | Flexitarian | Walk | Student | 1 |
| **MA Female 4** | 23 | 181 | 88.3 | 27.0 | 1.30 | 8.0 | 14,2 | Pescetarian | Walk | Student | 2 |
| **MA Female 5** | 30 | 171 | 77.8 | 26.8 | 1.28 | 8.4 | 14,3 | Vegetarian | Walk | Student | 2 |
| **HA Female 2** (activity) | 40 | 165 | 64.0 | 23.7 | 1.52 | 8.9 | 14,5 | Vegetarian | Car | Employed | 8 |
| **MA Female 6** | 35 | 152 | 76.3 | 33.0 | 1.65 | 8.3 | 14,5 | Pescetarian | Walk or bike | Student | 4 |
| **MA Female 7** | 38 | 167 | 59.8 | 21.4 | 1.69 | 8.8 | 14,7 | Flexitarian | Walk | Student | 1 |
| **MA Female 8** | 35 | 163 | 66.9 | 25.2 | 1.84 | 9.6 | 15,4 | Omnivore | Walk | Student | 3 |
| **HA Male 1** (runner) | 37 | 181 | 75.0 | 23.0 | 2.09 | 15.3 | 16,4 | Vegetarian | Walk or bike | Employed | 6 |
| **MA Female 9** | 23 | 162 | 61.8 | 23.6 | 1.40 | 8.7 | 16,5 | Vegetarian | Walk | Student | 2 |
| **MA Female 10** | 23 | 163 | 72.9 | 27.6 | 1.21 | 7.4 | 18,0 | Omnivore | Walk or bike | Student | 1 |
| **MA Male 1** | 31 | 182 | 74.9 | 22.6 | 1.54 | 10.5 | 18,0 | Omnivore | Walk or car | Student | 2 |
| **MA Female 11** | 24 | 170 | 62.2 | 21.5 | 1.60 | 6.9 | 19,7 | Omnivore | Car | Student | 2 |
| **MA Female 12** | 21 | 177 | 87.2 | 28.0 | 1.55 | 12.1 | 20,1 | Flexitarian | Car | Student | 2 |
| **MA Female 13** | 26 | 152 | 47.5 | 20.7 | 1.65 | 7.6 | 21,4 | Vegetarian | Car | Student | 1 |
| **HA Male 2** (runner) | 36 | 192 | 77.0 | 20.9 | 1.77 | 12.7 | 22,3 | Omnivore | Walk or home delivery | Employed | 2 |
| **MA Female 14** | 24 | 167 | 86.3 | 30.1 | 1.21 | 7.7 | 22,3 | Vegetarian | Car | Student | 3 |
| **MA Male 2** | 25 | 181 | 82.3 | 25.3 | 1.66 | 14.5 | 22,3 | Vegetarian | Walk or car | Student | 2 |
| **HA Female 3** (football) | 25 | 179 | 69.5 | 21.8 | 1.53 | 9.0 | 23,4 | Omnivore | Walk | Employed | 2 |
| **HA Male 3** (runner) | 26 | 182 | 70.3 | 21.2 | 1.62 | 14.0 | 23,4 | Omnivore | Walk | Student | 1 |
| **HA Male 4** (runner) | 31 | 190 | 64.9 | 18.0 | 2.56 | 14.7 | 25,3 | Flexitarian | Walk or bike | 50/50 | 2 |
| **MA Male 3** | 26 | 177 | 62.7 | 20.0 | 1.62 | 8.2 | 25,5 | Vegetarian | Walk or bike | Student | 2 |
| **MA Male 4** | 29 | 187 | 92.9 | 26.6 | 1.09 | 8.34 | 26,0 | Omnivore | Walk or bike | Student | 2 |
| **HA Female 4** (football) | 19 | 172 | 62.9 | 21.3 | 1.29 | 7.5 | 26,6 | Omnivore | No answer | Employed | 2 |
| **HA Female 5** (football) | 26 | 168 | 60.7 | 21.6 | 1.38 | 9.0 | 27,9 | Omnivore | Walk or bike | Employed | 1 |
| **MA Male 5** | 36 | 183 | 94.8 | 28.3 | 1.96 | 14.9 | 27,9 | Pescetarian | Walk or bike | Student | 1 |
| **HA Female 6** (CrossFit) | 31 | 167 | 67.2 | 24.1 | 2.01 | 10.9 | 28,5 | Flexitarian | Walk or car | Employed | 2 |
| **HA Male 5** (runner) | 32 | 194 | 76.9 | 20.4 | 2.21 | 14.8 | 28,5 | Omnivore | Walk | Parental leave | 3 |
| **HA Female 7** (runner) | 28 | 173 | 59.0 | 19.7 | 2.88 | 13.3 | 29,0 | Omnivore | Bike | Employed | 2 |
| **HA Male 6** (runner) | 35 | 184 | 79.4 | 23.5 | 1.97 | 13.1 | 30,3 | Vegetarian | Walk or bike | Employed | 3 |
| **HA Male 7** (runner) | 29 | 177 | 82.6 | 26.5 | 2.44 | 15.3 | 30,6 | Omnivore | Electric scooter | Student | 2 |
| **HA Female 8** (football) | 24 | 168 | 57.4 | 20.3 | 1.62 | 8.8 | 31,1 | Omnivore | Walk or bike | Employed | 2 |
| **HA Male 8** (runner) | 39 | 177 | 75.0 | 24.1 | 2.45 | 12.2 | 31,5 | Flexitarian | Home delivery | Employed | 2 |
| **HA Male 9** (runner) | 27 | 182 | 66.1 | 20.0 | 2.53 | 15.9 | 32,2 | Omnivore | Car | Employed | 2 |
| **HA Female 9** (runner) | 31 | 169 | 63.5 | 22.4 | 2.55 | 11.0 | 32,3 | Omnivore | Home delivery | Employed | 2 |
| **HA Male 10** (runner) | 28 | 182 | 70.4 | 21.4 | 2.31 | 11.9 | 33,1 | Omnivore | Car | Employed | 1 |
| **HA Male 11**(runner) | 25 | 190 | 80.5 | 22.3 | 1.93 | 13.7 | 33,5 | Omnivore | Walk, bike, home delivery | Employed | 2 |
| **HA Female 10** (football) | 29 | 173 | 63.0 | 21.1 | 1.74 | 10.5 | 36,8 | Omnivore | Car | Employed | 2 |
| **HA Male 12** (runner) | 23 | 189 | 71.9 | 20.1 | 1.90 | 14.0 | 37,0 | Omnivore | Walk or car | Student | 2 |
| **HA Female 11** (runner) | 26 | 169 | 60.9 | 21.3 | 1.52 | 10.5 | 39,9 | Omnivore | Car | Employed | 2 |
| **MA Male 6** | 20 | 175 | 70.5 | 23.0 | 1.61 | 10.2 | 39,9 | Omnivore | Walk | Student | 2 |
| **HA Male 13** (CrossFit) | 25 | 180 | 84.4 | 26.1 | 1.71 | 14.9 | 40,9 | Omnivore | Walk or bike | Employed | 2 |
| **MA Male 7** | 20 | 186 | 69.6 | 20.1 | 1.59 | 10.4 | 46,9 | Omnivore | Walk | Student | 1 |
| **HA Male 14** (runner) | 37 | 186 | 79.5 | 23.0 | 1.93 | 15.8 | 48,9 | Flexitarian | No answer | Employed | 1 |
| **MA Male 8** | 31 | 188 | 148.8 | 42.3 | 1.23 | 12.4 | 84,9 | Omnivore | Walk | Employed | 2 |
|  |  |  |  |  |  |  |  |  |  |  |  |

BMI, Body Mass Index; EI, Energy Intake

| **Supplemental Table 3.** Total energy intake, macronutrient distribution, and carbon dioxide equivalents are grouped by both gender and activity level. | | | | | | | | | |
| --- | --- | --- | --- | --- | --- | --- | --- | --- | --- |
|  | | | Moderately-active women (n=14) | | Moderately-active men (n=8) | Highly-active women (n=11) | Highly-active men (n=14) |  |  |
|  | | | **Mean** (SD) | | **Mean** (SD) | **Mean** (SD) | **Mean** (SD) | **p-value** |  |
| Total energy intake (kcal) | | | 2092 (371) | | 2671 (606) | 2347 (379) | 3385 (312) | <0.001* |  |
| Total energy intake (MJ) | | | 8.8 (1.6) | | 11.2 (2.5) | 9.8 (1.6) | 14.2 (1.3) | <0.001* |  |
| Carbohydrate intake (g/d) | | | 229.7 (51.9) | | 268.0 (67.3) | 262.4 (46.0) | 373.2 (39.9) | <0.001* |  |
| Carbohydrate E% | | | 46.3 (4.7) | | 42.6 (6.5) | 46.8 (4.5) | 47.0 (4.8) | 0.179 |  |
| Fat intake (g/d) | | | 87.9 (22.9) | | 121.9 (37.6) | 83.4 (27.9) | 139.6 (28.0) | <0.001* |  |
| Fat E% | | | 38.2 (4.5) | | 41.0 (5.0) | 36.7 (5.7) | 32.8 (6.9) | 0.012* |  |
| Fibre intake (g/d) | | | 28.1 (12.2) | | 25.5 (11.7) | 32.1 (7.1) | 37.6 (8.0) | 0.029* |  |
| Fibre (g/MJ) | | | 3.1 (1.0) | | 2.2 (0.6) | 3.2 (0.7) | 2.6 (0.5) | 0.011* |  |
| Protein intake (g/d) | | | 69.6 (15.5) | | 91.6 (16.7) | 119.6 (32.8) | 124.4 (26.6) | <0.001* |  |
| Protein g/kg body weight | | | 1.0 (0.2) | | 1.1 (0.2) | 1.9 (0.6) | 1.7 (0.4) | <0.001* |  |
| Protein E% | | | 14.2 (2.4) | | 12.6 (2.2) | 14.9 (2.9) | 19.3 (6.8) | 0.003* |  |
| Plant-based protein (g/d) | | | 34.3 (11.7) | | 38.5 (19.5) | 44.8 (13.2) | 55.4 (11.5) | <0.001* |  |
| Animal-based protein (g/d) | | | 33.7 (14.4) | | 54.6 (11.7) | 72.4 (35.5) | 64.9 (27.9) | <0.001* |  |
| Co_2_e/day (kg) | | | 2.3 (0.6)3 | | 5.2 (3.1) | 3.9 (1.2) | 4.4 (1.2) | <0.001* |  |
| Co_2_e/MJ (kg) | | | 0.27 (0.09) | | 0.47 (0.25) | 0.39 (0.12) | 0.31 (0.08) | 0.005* |  |
|  |  |  | |  |  |  |  |  |  |

MJ, Mega Joule; kcal, kilo calories; Co_2_e, carbon dioxide equivalent

**Comparisons between groups tested with One-way ANOVA, p-values <0.05 was considered significant. Significant findings were tested with the Bonferroni post hoc test*
